# Case report: isolation of *Hydrogenophaga* from septic blood culture following near-death drowning in lakewater

**DOI:** 10.1099/acmi.0.000533.v4

**Published:** 2023-09-19

**Authors:** Stuart Feichtinger, Angela A. Lazar, Megan A. Luebbe, Molly A. Accola, Brittney D. Jung-Hynes, Patti J. Anderson, Kelly M. Koglin, Karen S. Schliesman, William Ehlenbach, Jeannina Smith, Derrick J. Chen, William M. Rehrauer, Adam L. Bailey

**Affiliations:** ^1^​ Department of Anesthesiology, Division of Critical Care, University of Wisconsin–Madison School of Medicine and Public Health, Madison, WI, USA; ^2^​ University of Wisconsin Hospitals and Clinics, Clinical Laboratories, Madison, WI, USA; ^3^​ Department of Medicine, University of Wisconsin–Madison School of Medicine and Public Health, Madison, WI, USA; ^4^​ Department of Pathology and Laboratory Medicine, University of Wisconsin–Madison School of Medicine and Public Health, Madison, WI, USA

**Keywords:** 16S sequencing, drowning, hydrogenophaga, MALDI-TOF

## Abstract

A patient suffered a non-fatal wet drowning in a freshwater lake and developed bacteraemia several days later. Blood culture grew a Gram-negative rod that could not be identified by matrix-assisted laser desorption/ionization time-of-flight mass spectrometry (MALDI-TOF MS). 16S ribosomal RNA sequencing of the isolate identified the microbe as *Hydrogenophaga laconesensis* – an environmental microbe commonly found in freshwater. The recovery of multiple pathogenic micro-organisms (although not *H. laconesensis *) from culture of respiratory specimens prompted the initiation of antibiotic therapy with cefepime and, later, vancomycin. The patient’s clinical course gradually improved over the course of 2 weeks and she was ultimately discharged home with minimal sequelae. To our knowledge, this is the first evidence of human infection with bacteria in the genus *Hydrogenophaga *. *Hydrogenophaga* may be considered in cases of freshwater near-drowning, and MALDI-TOF MS databases should be updated to include *H. laconesensis *.

## Data Summary

Matrix-assisted laser desorption/ionization time-of-flight mass spectrometry (MALDI-TOF MS) was performed on the Biotyper 4.1 (Bruker Daltonik) using the MBT 8468 database. 16S rRNA was perfromed on the ABI 3500xL (Thermo Fisher) and analysis was performed using SmartGene IDNS v3_8_1r1(r31140). Sequencing reads have been deposited under GenBank accession numbers OP851759–OP851762.

## Introduction

The genus *Hydrogenophaga* consists of motile Gram-negative, rod-shaped, yellow-pigmented bacteria commonly found in association with freshwater [1, 2]. A small number of strains have been studied for their unique enzymatic properties, including the namesake ability to oxidize hydrogen [3, 4]). Although viable *Hydrogenophaga* bacteria are often found in drinking water [2, 5], no reports of human infection with bacteria from this genus have been reported.

## Case description

A previously healthy adolescent female dived into a shallow lake in the Upper Midwest region of the USA during the summer. She failed to resurface and was found floating face down in the water approximately 5 min later. She was minimally responsive upon being pulled from the water. Upon arrival of emergency medical services, fluid was observed in her pharynx and prominent ronchi were heard upon chest auscultation. Blood oxygen saturation was low (80–85 %; reference range: 92–100 %) despite 100 % FiO_2_ by bag–valve–mask ventilation. Intubation was attempted twice by ambulance personnel, but was unsuccessful. A supraglottic airway (i-gel, Intersurgical) was placed, but removed shortly after due to aspiration concerns when the patient began vomiting a large volume of liquid consistent with lake water. Following this, a third attempt at intubation by ambulance personnel failed as well and her respirations were again supported with bag–valve–mask ventilation. An aircraft with the regional Helicopter Emergency Medical Service (HEMS) arrived on scene, and HEMS physicians were able to successfully intubate the patient via video laryngoscopy, noting a significant amount of frothy secretions in her airway. She was transferred by helicopter to the University of Wisconsin Hospital. Of note, she was given a full-body chlorhexidine scrub-down upon admission. Radiological evaluation revealed extensive, predominantly centrally located, ground glass and consolidative opacities in both lungs concerning for large-volume aspiration. She was admitted to the intensive care unit (ICU) with a diagnosis of severe acute respiratory distress syndrome (ARDS) secondary to submersion injury. Her PaO_2_/FiO_2_ (P/F) ratio on admission was 70 (reference range: normal>=400, severe ARDS <100) despite a positive end-expiratory pressure (PEEP) of 18 cm of water (reference range: 5–10 cm). Approximately 10 h after her drowning incident, the patient became febrile (38.4 °C; reference range: 36.2–37.5 °C) with leukocytosis to 13.4 thousand cells µl^−1^ (reference range: 3.8–10.5 K µl^−1^) and developed progressive vasodilatory shock despite significant doses of norepinephrine (0.3 mcg kg^−1^ min^−1^) and vasopressin (0.03 units min^−1^). This prompted collection of two blood culture sets (one by venipuncture of the left hand and one via a an existing right internal jugular central venous catheter), sputum and urine cultures, followed by initiation of cefepime for empirical coverage of common freshwater pathogens, including *Pseudomonas, Aeromonas* and *Proteus *.

## Diagnostic assessment

**Fig. 1.**
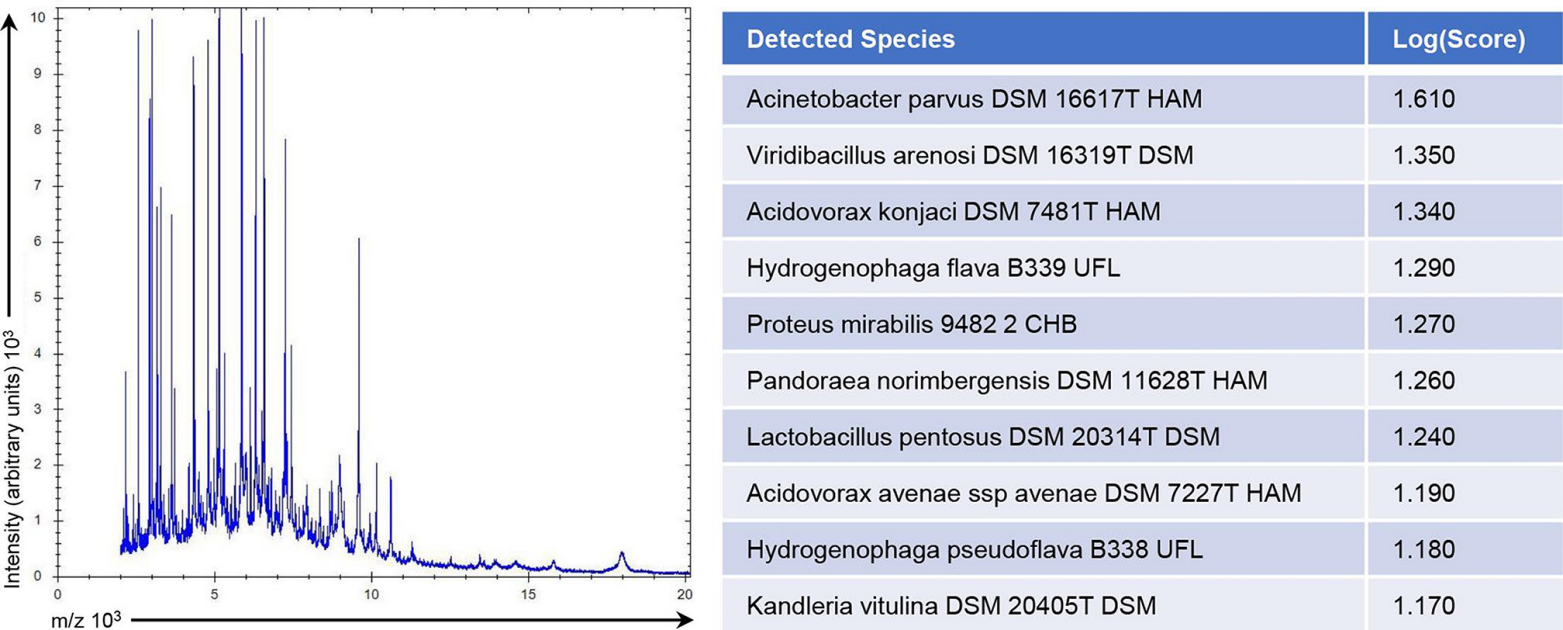
Mass spectrum profile of *Hydrogenophaga laconesensis* and rank-ordered matches against the Bruker MALDI-TOF MS MBT 8468 database.

**Fig. 2.**
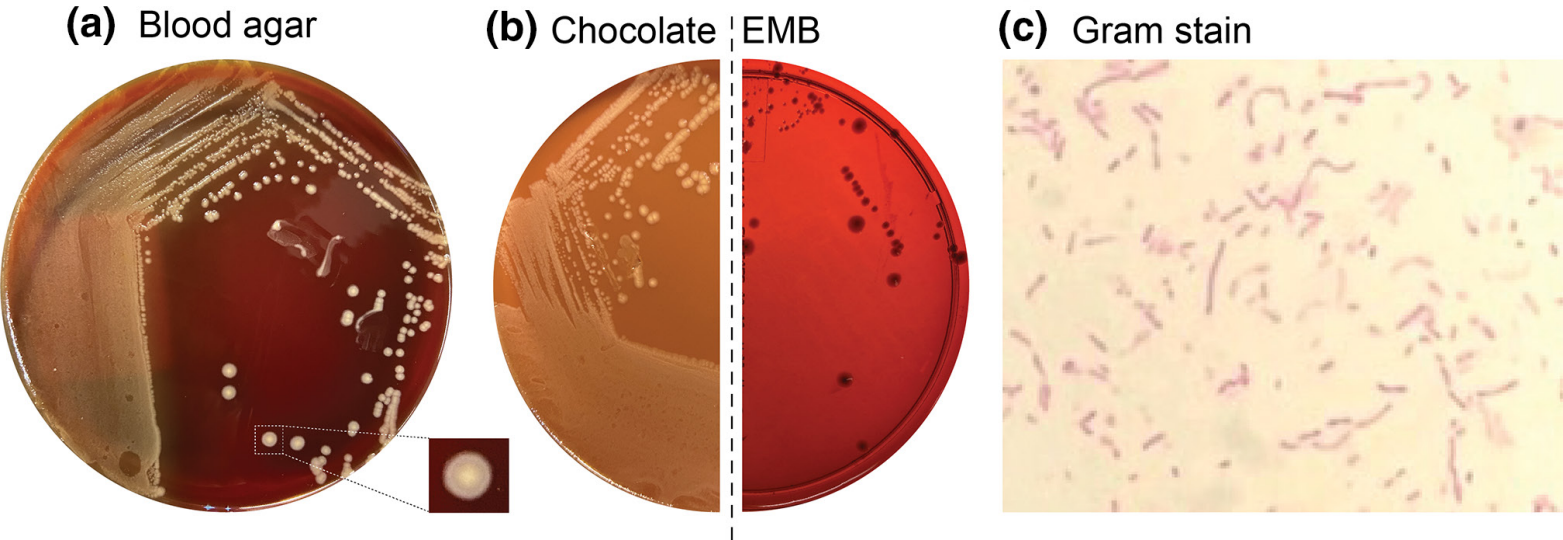
Growth characteristics of *Hydrogenophaga laconesensis* on various culture media, isolated from near-death drowning victim.

Culture of respiratory specimens grew *Aeromonas* sp., *Streptococcus pneumoniae *, *Staphylococcus aureus* and endogenous flora at various points throughout the patient’s hospital stay. Separately, one of two aerobic blood culture bottles (BACTEC, BD) – the specimen collected from the patient’s left hand – flagged as positive on day 2 of blood culture (day 3 of hospitalization) and was subjected to direct-from-specimen matrix-assisted laser desorption/ionization-time-of-flight mass spectrometry (MALDI-TOF MS) (MALDI Biotyper 4.1, Bruker Daltonik, MBT 8468 database). A strong mass spectrum profile was generated for each MALDI-TOF MS attempt, but no high-confidence matches were identified (Fig. 1). Sub-culture yielded a pure culture of non-haemolytic cream-coloured colonies on sheep blood agar after 24 h (Fig. 2a). Growth was also present on chocolate and eosin methylene blue (EMB) agar (Fig. 2b), with Gram stain showing a Gram-negative rod (Fig. 2c). However, this isolate repeatedly failed multiple attempts at identification by MALDI-TOF MS. DNA extraction (Qiagen), 16S rRNA gene real-time PCR (Roche, LC 480), DNA sequencing (Thermo Fisher, ABI 3500xL) and alignment [SmartGene IDNS v3_8_1r1(r31140)] identified a match to *Hydrogenophaga laconesensis* with 99 % identity in both 5′ and 3′ reads (GenBank accession numbers OP851759–OP851762), forming a contig that contained zero mismatches to reference strain KT756664 across 1444 nucleotides. The concurrent aerobic blood culture (from the right internal jugular) remained negative. Antibiotic susceptibility testing (NM56 panel, MicroScan, Beckman Coulter) on the isolate proved unreliable and was not reported or used for clinical decision-making.

## Clinical course

**Fig. 3.**
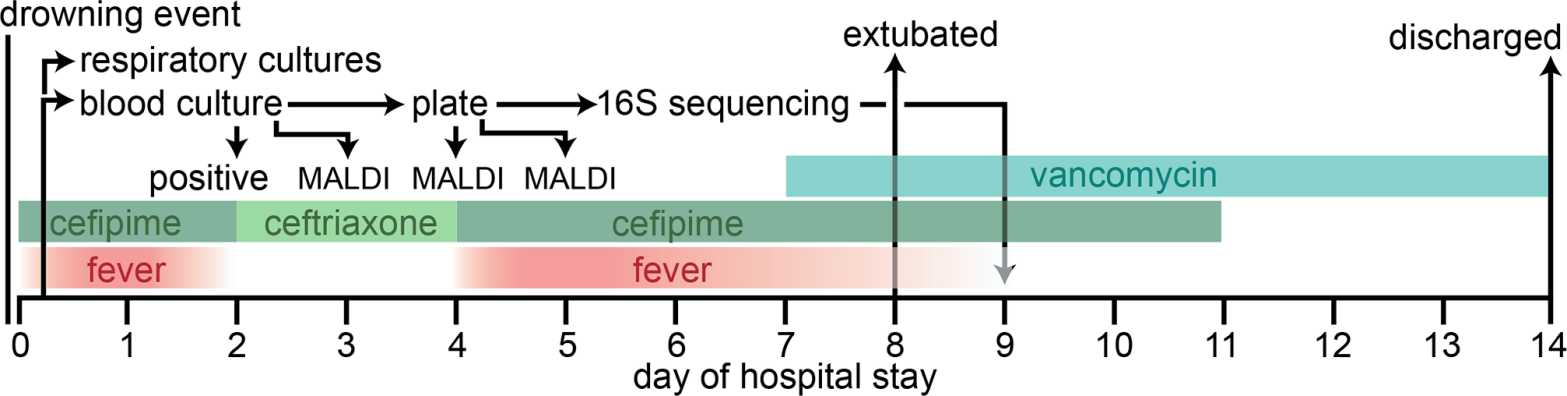
Timeline of clinical course and microbiological workup pertinent to the identification of *Hydrogenophaga laconesensis *.

The patient’s fever, leukocytosis and vasopressor requirement began to improve on cefepime therapy, with the P/F ratio also improving to 200 (i.e. moderate ARDS). On hospital day 3 her antibiotic therapy was narrowed to ceftriaxone. However, her fever returned on hospital day 4, prompting reinitiation of cefepime for an additional 7 day course. Additional blood cultures drawn at this time remained negative. Over the next several days, her fever resolved and her sedation was gradually weaned. She was found to be completely neurologically intact and able to communicate with her care team by writing. Despite her significant overall improvement, her ventilatory and oxygen requirements remained high with progressive bilateral opacities on chest X-ray and multiple failed spontaneous breathing trials (SBTs). Bronchoscopy with bronchoalveolar lavage was performed on hospital day 7 with recovery of endogenous flora and *Corynebacterium striatum *, prompting initiation of vancomycin therapy. Her respiratory status improved markedly over the next 24 h and she was extubated on hospital day 8, with supplemental oxygen provided by nasal cannula. She was transferred out of the ICU on hospital day 10 and discharged home on room air on hospital day 15 following completion of a 7 day course of vancomycin. Several weeks later, the patient was contacted through the secure messaging portal in her electronic medical record and provided her consent for this case report. A timeline of the patient’s clinical course and microbiological workup pertinent to the identification of *H. laconesensis* can be found in Fig. 3.

## Discussion

To our knowledge, this is the first case of human infection with *Hydrogenophaga *. It seems likely that this organism was present in the lakewater inhaled by the patient given the affinity of *Hydrogenophaga* species for freshwater and the mechanism of the patient’s lung injury. Although *H. laconesensis* was not recovered from respiratory specimens, this could potentially be explained by low abundance of this organism in the respiratory specimens collected; anatomical variation in specimen collection; out-competition by other bacteria; or mistaking the growth of *H. laconesensis* colonies for endogenous flora. It is also possible that *H. laconesensis* was present on this patient’s skin and introduced into the blood culture bottle via venipuncture, although the chlorhexidine scrub-down the patient received on admission plus the standard venipuncture site preparation make this less likely. Thus, the route by which *H. laconesensis* entered the patient’s bloodstream; whether this bacteria established a nidus of infection in the lung; and the contribution of pulmonary *H. laconesensis* infection to ARDS severity, remain open questions. However, the pure culture of *H. laconesensis* leaves little doubt that bacteraemia could have been caused by a second co-infecting organism. The impact of cefepime and vancomycin therapy on *H. laconesensis* infection also remains unknown, although the patient did improve while on these antibiotics and subsequent blood cultures were negative.

Three species within the genus *Hydrogenophaga* are included in the Bruker MALDI-TOF MS MBT 8468 database: *H. flava *, *H intermedia* and *H. Pseudoflava*. MALDI-TOF MS analysis of colonies from the blood agar plate identified *H. flava* and *H. pseudoflava* as potential matches (rank #4 and #9, respectively), but log scores were of low confidence (1.290 and 1.180, respectively) (Fig. 1).

In conclusion, *Hydrogenophaga* infection may be considered in cases of freshwater near-drowning, and updating MALDI-TOF MS databases to include *H. laconesensis* may be helpful for identification.
